# Clutter Mitigation in Indoor Radar Sensors Using Sensor Fusion Technology

**DOI:** 10.3390/s25103113

**Published:** 2025-05-14

**Authors:** Srishti Singh, Ha-Neul Lee, Yuna Park, Sungho Kim, Si-Hyun Park, Jong-Ryul Yang

**Affiliations:** 1 Department of Electronics Engineering, Yeungnam University, Gyeongsan 38541, Republic of Korea; srishtisingh18@yu.ac.kr (S.S.); sunghokim@yu.ac.kr (S.K.); sihyun_park@yu.ac.kr (S.-H.P.); 2 Department of Electrical and Electronics Engineering, Konkuk University, Seoul 05029, Republic of Korea; hnlee96@konkuk.ac.kr (H.-N.L.); engle0108@konkuk.ac.kr (Y.P.)

**Keywords:** clutter mitigation, millimeter-wave radar sensor, low-resolution camera, sensor fusion, signal-to-noise ratio

## Abstract

A methodology utilizing low-resolution camera data is proposed to mitigate clutter effects on radar sensors in smart indoor environments. The proposed technique suppresses clutter in distance–velocity (range–Doppler) images obtained from millimeter-wave radar by estimating clutter locations using approximate spatial information derived from low-resolution camera images. Notably, the inherent blur present in low-resolution images closely corresponds to the distortion patterns induced by clutter in radar signals, making such data particularly suitable for effective sensor fusion. Experimental validation was conducted in indoor path-tracking scenarios involving a moving subject within a 10 m range. Performance was quantitatively evaluated against baseline range–Doppler maps obtained using radar data alone, without clutter mitigation. The results show that our approach improves the signal-to-noise ratio by 2 dB and increases the target detection rate by 8.6% within the critical 4–6 m range, with additional gains observed under constrained velocity conditions.

## 1. Introduction

The comparison of millimeter-wave radar sensors with high-resolution cameras, thermal sensors, and infrared sensors highlights their potential for the accurate detection of moving objects in indoor environments. Unlike other sensors, millimeter-wave radar ensures stable detection performance regardless of environmental conditions or illumination, while preserving privacy. These advantages make it well suited for real-time continuous monitoring in smart indoor environments [1,2].

Despite the numerous advantages of radar-based smart environmental monitoring, multipath scattering and ghost targets caused by stationary clutter remain major obstacles to reliable detection performance in typical indoor settings [3,4]. In indoor environments, clutter primarily arises from strong static reflections from walls, floors, ceilings, and furniture. These reflections often dominate the radar scene and significantly overlap with the returns from actual moving targets in the range–Doppler (RD) domain. This problem becomes particularly severe when the target moves slowly or pauses momentarily, as the Doppler signatures become indistinguishable from those of static clutter. Conventional signal processing techniques, such as static clutter suppression and moving target indication (MTI), offer only limited effectiveness in these cases. Specifically, such methods may inadvertently remove or distort low-velocity targets along with the clutter. Additionally, multipath reflections further degrade spatial localization accuracy, leading to false alarms and target ambiguity. These factors collectively underscore the complexity of clutter suppression in indoor radar sensing. Mitigating the effects of clutter has long been recognized as a fundamental challenge in radar signal processing and has been the subject of extensive research. Among various approaches, sensor fusion methods, particularly those that combine radar with complementary modalities, have proven effective in reducing clutter effects by lowering signal processing complexity [5]. Moreover, these techniques enhance detection accuracy by combining multisensory data to extract meaningful information [6]. Radar-only methods use micro-Doppler and range–Doppler features to distinguish moving from stationary targets, but may be less effective in cluttered indoor settings with slow or partially occluded subjects, where spatial cues from cameras offer added benefit [7,8].

Most existing object detection methods employing sensor fusion integrate radar-generated data primarily to complement camera-based object recognition, relying heavily on visual identification from high-resolution images [9,10,11,12,13]. However, such approaches require complex processing and substantial data handling capacity, significantly increasing system cost and complexity [14,15]. Crucially, they also undermine radar’s inherent privacy advantages, as initial object identification depends on visual data from cameras, which can potentially expose sensitive image data to security vulnerabilities [16,17]. In contrast, millimeter-wave radar inherently addresses these limitations, ensuring consistent and reliable detection under varying illumination and environmental conditions, and avoiding privacy concerns associated with visual data storage or preprocessing. Thus, from a system-design perspective, prioritizing radar data as the primary information source is both logically consistent and practically advantageous. Consequently, our approach emphasizes radar data processing, leveraging complementary spatial information from low-resolution cameras solely to enhance radar clutter suppression, without compromising radar’s inherent privacy and robustness advantages.

In this study, we propose a lightweight sensor fusion methodology to mitigate clutter in millimeter-wave radar-based indoor monitoring. The approach integrates coarse spatial cues, such as bounding box location and persistence, obtained from a low-resolution monocular camera. Using low-resolution imagery preserves user privacy, lowers computational demands, and avoids the complexity of image-level fusion. Only minimal spatial context is required, enabling effective clutter identification without the need for high-fidelity visual data. The proposed pipeline consists of independent sensing, clutter region estimation, and radar-domain masking guided by camera-derived information. This fusion process enhances the signal-to-noise ratio (SNR) and improves in-range detection performance, while maintaining real-time processing capability. The proposed method enables scalable and practical deployment in smart environments using cost-effective, privacy-aware hardware.

## 2. Methodology

A commonly adopted strategy for radar–camera fusion combines images produced by both sensors, which demands considerable computational resources and requires radar waveforms to be converted into image form [18]. Such an operation can further intensify system requirements in real-time processing. In contrast, the method proposed here circumvents the influence of clutter on radar sensors by implementing a straightforward masking mechanism that obscures clutter regions in the camera-captured image. By recording only the approximate location of the clutter at a low resolution, the camera mitigates privacy concerns often associated with high-fidelity imaging. Moreover, the proposed technique effectively eliminates only the clutter regions that degrade radar detection performance, thus obviating the need for direct image-domain fusion of heterogeneous sensor data. In doing so, it sidesteps the inherent domain mismatch constraints of conventional image-level fusion approaches, reducing both computational overhead and privacy risks while maintaining robust detection capabilities. Each sensor modality (radar and camera) plays a distinct role in the proposed fusion process: the radar delivers reliable range and velocity information, while the camera provides coarse spatial localization of clutter. By integrating these complementary cues, the masking operation becomes both spatially guided and computationally efficient. Unlike conventional filtering methods, our approach embeds cross-modal information directly into the radar domain without relying on deep learning or image-level processing, thereby enhancing clutter suppression while preserving real-time feasibility.

**Figure 1:**
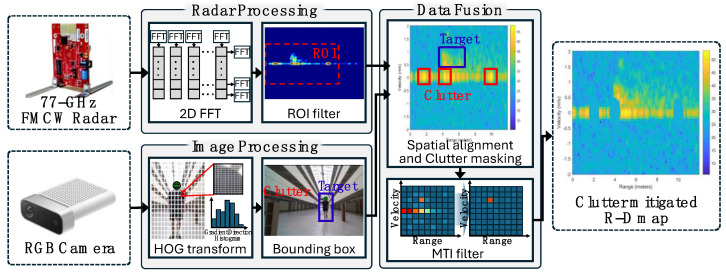
Signal processing procedure for the proposed data fusion.

Figure 1 illustrates the proposed signal processing procedure along with the experimentally acquired data. The beat frequency information obtained via a frequency-modulated continuous-wave (FMCW) radar provides the range of objects within the radar antenna’s field of view, whereas the Doppler frequency reveals the velocity of the objects [19,20,21]. A range–Doppler (RD) map, which jointly represents the objects’ range and velocity, is particularly useful for understanding the state of all objects within the radar field of view at a specific moment in time [22,23]. In an indoor environment with limited space, the object velocity is inherently restricted. When narrowing the velocity coverage on the RD map to reflect these constraints, noise introduced by stationary clutter becomes dominant due to the inherent range and velocity resolution of the radar. Strong scattering from stationary clutter can substantially degrade the SNR, hindering target detection in the RD map. Simultaneously, a low-resolution camera detects objects by identifying abrupt changes in RGB (red, green, and blue) levels between the background and the potential target. Each detected object is represented by a bounding box for size estimation, and a rough distance is inferred by a triangulation method, comparing the camera’s known field of view with the bounding box dimensions [24,25]. This method does not rely on stereo vision or depth sensing, but instead leverages geometric projection assumptions to estimate distance from object scale within the image. While the accuracy of this distance estimation can depend on the camera resolution—and thus remains lower than that of time-of-flight sensors—it can exceed the distance resolution provided by the radar. Although synchronizing radar and camera data is crucial for overall consistency in the conventional sensor fusion technique, the requirement for synchronization is not significant in the proposed technique. Consequently, synchronization errors have a negligible impact on stationary clutter removal, where the scene remains unchanged over time.

The camera employs a pre-trained detection model to accurately identify and locate both target objects and surrounding clutter in the scene. Specifically, a histogram of oriented gradients (HOG) model is utilized to detect human figures, generating bounding boxes around identified individuals [26,27]. These boxes are then dynamically scaled and calibrated according to the camera’s sensing characteristics to ensure precise representation in the radar coordinate framework. Subsequently, these calibrated bounding boxes are approximately aligned with the radar’s spatial coordinate system, utilizing estimated range values. Given the inherent limitations of camera sensors in providing accurate depth measurements, a coarse distance estimate is derived from the height of the bounding box relative to the known field-of-view parameters of the camera, with a maximum error of approximately ±0.5 m for objects located at a distance of 5 m. This coarse distance metric is associated with corresponding range bins within the radar’s RD map. This process facilitated the approximate projection of clutter regions into the radar domain, as only range alignment was essential for the clutter mitigation approach proposed in this study. To address segmentation errors encountered in low-resolution imagery, morphological operations are applied, thereby creating more coherent bounding regions. Notably, the inherent blur of low-resolution camera images naturally aligns with the distortion patterns induced by clutter in radar signals, making them particularly suitable for clutter estimation [28]. To distinguish moving targets from static clutter, bounding boxes exhibiting motion across consecutive frames captured by the static camera are classified as target objects, whereas those that remain stationary are labeled as clutter. In parallel, the FMCW radar determines the distance via the beat frequency of the baseband signals and velocity via the Doppler frequency. For example, these frequencies can be derived by applying a Fast Fourier Transform (FFT) to 9.8 million samples collected at a 5 kS/s sampling rate, generating a frame-by-frame RD map over time. Both the camera and the radar are triggered simultaneously by a single control signal from the PC to minimize fusion errors. Because the target’s movement is relatively slow compared to the data acquisition interval, complex compensation techniques for synchronizing the two data streams are deemed unnecessary. Following detection, camera-derived bounding boxes are mapped onto the radar’s RD domain, aligning the camera’s field of view with the radar’s spatial resolution. Although FMCW radar systems offer reliable range and velocity information, their resolution is fundamentally constrained by available bandwidth, making it difficult to isolate small or closely spaced objects. In indoor environments, strong reflections from static clutter sources, such as furniture or walls, can obscure genuine targets even without inducing Doppler shifts. Conversely, low-resolution monocular cameras provide only approximate spatial cues, typically in the form of bounding boxes. While insufficient on their own for absolute localization, these cues offer valuable spatial references. In our proposed fusion strategy, the relative positions of static clutter are extracted from the camera image and aligned with radar range bins to spatially mask these regions. This guided masking enhances radar performance without relying on high-fidelity vision or complex data fusion. The use of coarse camera cues, when paired with radar’s Doppler sensitivity, facilitates the targeted suppression of persistent static clutter and supports reliable moving target detection in constrained environments. Regions identified as clutter by the camera are superimposed on the RD map, and their corresponding radar intensity values are set to zero, effectively suppressing static reflections while retaining potential moving targets. To further refine the distinction between stationary and moving objects, a moving target indication (MTI) filter is employed to eliminate residual static components. Static clutter, by definition, does not exhibit Doppler shift and hence appears near the zero-velocity region in the range–Doppler map. Traditional radar signal processing methods like MTI filtering can suppress such stationary signals, but their performance is often limited in environments with multipath reflections or partially moving objects. The low-resolution camera used in this study does not aim to detect Doppler shifts; instead, it localizes persistent spatial clutter that typically remains unresolved by MTI filtering due to its non-Doppler nature or spatial complexity. By incorporating these approximate spatial cues from the camera into the radar domain, our fusion method enables more targeted and robust clutter suppression beyond what is possible with radar-only techniques.

To mitigate the impact of undesired reflections and stationary objects, additional clutter-removal techniques are proposed. These include circular or square region removal, as well as adaptive masking based on known clutter locations. In the camera data, clutter does not remain at a fixed position from frame to frame, owing to factors such as camera resolution, field of view, incoming noise, and the accuracy of the pre-trained detection model. Consequently, the clutter location exhibits a distribution of errors instead of remaining constant. The proposed method exploits this distribution to define the size of the clutter region to be removed, which is sufficiently smaller than the radar resolution and thus directly applicable for clutter suppression in the RD map. However, because radar-based clutter often appears larger than its actual physical extent due to electromagnetic scattering and diffraction, the clutter removal process dynamically adjusts the shape and size, typically squares or circles, of the masking region. This dynamic compensates for the difference between the camera-based clutter estimate and the more extensive radar echoes, thereby ensuring the robust mitigation of static clutter in integrated camera–radar imaging.

## 3. Results and Discussion

### 3.1. Measurement Environment

**Figure 2:**
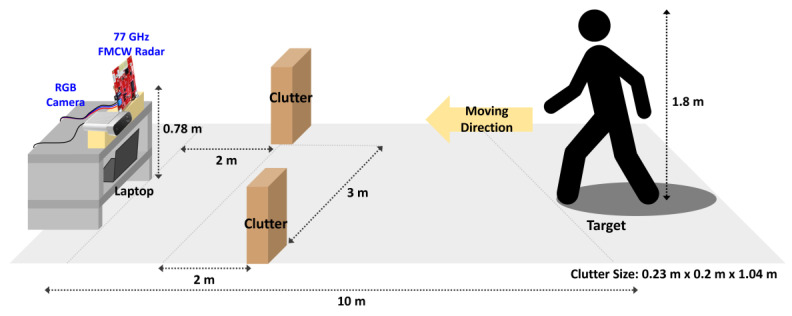
Diagram of measurement environment for validation of the proposed radar–camera sensor configuration.

To validate the effectiveness of the proposed radar–camera sensor configuration for clutter mitigation, an experiment was conducted in an indoor environment that included clutter while minimizing multipath effects, as shown in Figure 2. The clutter source consisted of two wooden bookcases measuring 1.04 m in height and 235 mm in width. Because the ceiling and side walls were located at least 20 m away from the sensor module, the radar’s field of view was not obstructed, thereby minimizing multipath contributions from these surfaces. A subject with a height of 1.8 m was positioned 10 m from the integrated sensor module and walked back and forth along the line of sight to the module. The subject’s speed and distance were measured using a Texas Instruments 77 GHz FMCW radar sensor module, configured with a 2 GHz frequency bandwidth, a pulse repetition time of 0.2 ms, a frame rate of 39 fps, and a fixed field of view of ±60°. These parameters were selected to ensure accurate target detection and to facilitate subsequent clutter-removal analysis. An Azure Kinect camera, operating at 30 fps with a resolution of 1280 × 720 and a fixed horizontal view angle of 90°, was used to capture visual data in the experimental environment. To mitigate the influence of non-line-of-sight (NLoS) effects through the floor, the integrated module was mounted on a cart at a height of 0.78 m above ground level. All sensor data were captured by a single personal computer (PC), which included a unified control function to initialize both sensors simultaneously. The data streams were logged at a high rate (≥30 fps) relative to the subject’s walking speed, thereby minimizing synchronization errors arising from time offsets between sensor activation and data collection.

### 3.2. Experimental Results from Measuring Distance and Velocity of a Moving Subject

**Figure 3:**
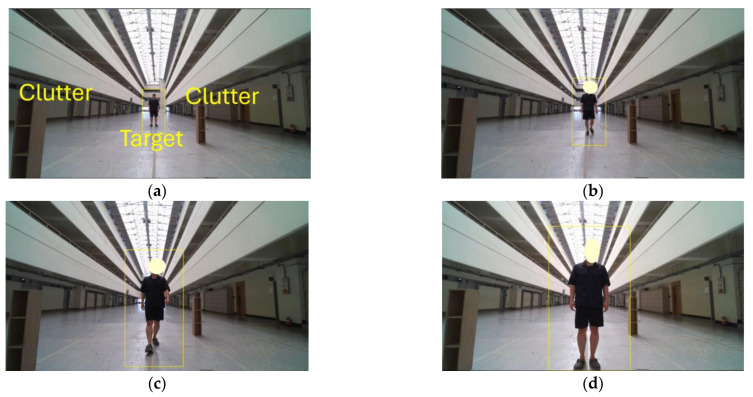
Experiment photographs of a walking subject in the environment with clutter.

Figure 3 shows photographs of the experiment performed by a subject moving forward in the environment shown in Figure 2. As described in Section 2, the beat and Doppler frequencies obtained from the radar signals can be mapped to distance and velocity, respectively, thus forming a frame-by-frame RD matrix. The RD map is subsequently generated by including the power, expressed in dB, derived from the magnitude of the frequency-domain representation via FFT. Due to experimental constraints, the detection range was limited to 0~12 m, and the velocity range was restricted to −2~2 m/s to accommodate the subject’s gait and back-and-forth motion. These limitations diminish both the coverage and the resolution of the RD map, thereby amplifying the influence of clutter. Figure 3a–d sequentially illustrate the subject approaching the sensor from a distance, capturing changes in the relative position over time. 

**Figure 4:**
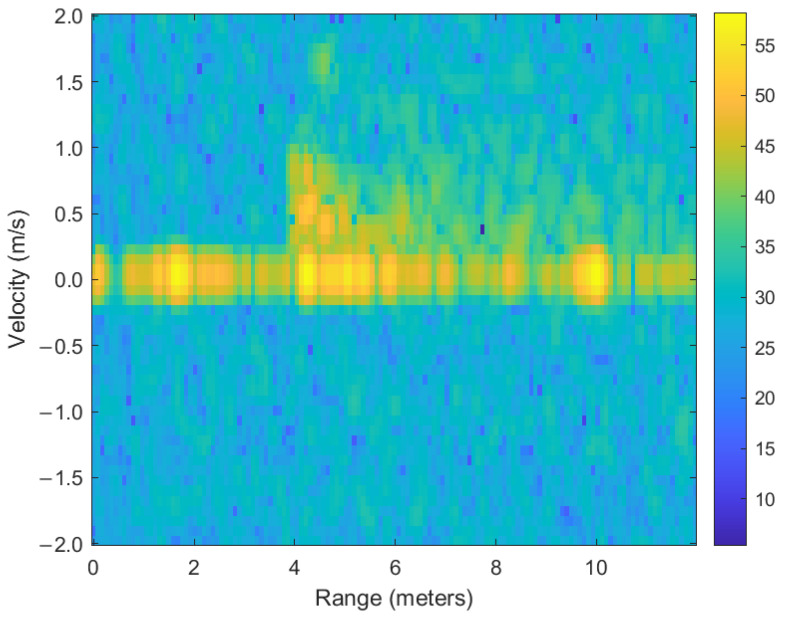
Measured range–Doppler map for the walking subject in the experiment environment.

As depicted in Figure 4, the radar sensor captured an RD map of a subject traversing the sensor’s field of view. The clutter sources at 2 m and 4 m exhibited the strongest signal power, remaining unchanged regardless of the subject’s motion. In addition to these intentionally placed clutter sources, another clutter source was observed at 10 m in the measured RD map. As shown in Figure 3, this artifact may be attributed to environmental factors such as the ceiling height or corridor walls on a different floor, thereby contributing additional clutter. While these clutter sources manifest high radar cross-section (RCS) reflections concentrated at discrete points, the subject’s signal appears more diffusely distributed due to the independent movements of the torso, arms, and legs, and consequently exhibits comparatively lower reflected power. As illustrated by the measurement results in Figure 4, clutter emerges as a dominant factor in the SNR degradation during the extraction of the subject’s trajectory.

### 3.3. Clutter Mitigation in the Proposed Sensor Configuration

#### 3.3.1. Implementation of Signal Processing

**Figure 5:**
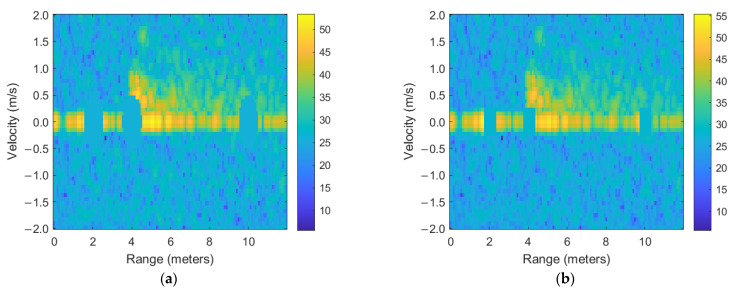
Range–velocity map comparison: (**a**) map for masked circular regions; (**b**) map for masked square regions.

In the RD map illustrated in Figure 4, clutter does not appear as a singular discrete point, but rather occupies a continuous region within the two-dimensional radar image, represented by an array with dimensions of 128 (velocity bins) × 256 (range bins). This extended clutter area is fundamentally defined by the radar system’s inherent range and velocity resolutions. Specifically, stationary clutter typically manifests as a distinct section centered around the zero-velocity axis, with its exact location and extent influenced by the physical environment and radar characteristics. Given the clutter location information provided by a co-located camera sensor and considering the specific structural attributes of the RD map, we implemented a targeted clutter removal procedure by strategically applying masking techniques directly onto the RD map. The masks used in this process were individually designed in two primary geometric forms, circular and square, each centered precisely around the clutter’s dominant range coordinates identified by the camera data at the zero-velocity position. When determining the optimal size and shape of these masks, special consideration was given to the inherent resolution disparities between the range and velocity dimensions within the RD map. Generally, clutter at zero velocity is significantly influenced by velocity resolution due to the use of MTI filters, which primarily differentiate stationary from dynamic objects based on their Doppler characteristics. Hence, the dimensions of the circular and square masking areas were established according to the larger of the two resolution values (either range or velocity). In most scenarios involving stationary clutter suppression, the velocity resolution typically represents the limiting factor due to the necessity of precisely isolating the zero-velocity clutter components. Through this careful mask definition and strategic application guided by multimodal sensing information, the implemented clutter removal method effectively suppresses unwanted stationary targets, thereby significantly enhancing the radar’s detection and imaging performance for dynamic targets in complex operational environments. Figure 5 shows the processed images with the proposed clutter mitigation technique for the measured RD map.

#### 3.3.2. Improvements with the Proposed Configuration

The effectiveness of the proposed clutter removal strategy was quantitatively assessed by comparing the SNR across three distinct datasets: unprocessed radar data, radar data processed using circular-region masking, and radar data processed with square-region masking. Initially, the raw RD map exhibited an SNR of approximately −20.7 dB, clearly indicative of significant interference from stationary clutter predominantly concentrated around the zero-velocity region. Upon applying circular-region masking, which strategically targets the localized clutter areas identified via radar–camera fusion, the SNR improved substantially to −18.7 dB. This 2 dB increase demonstrates a meaningful attenuation in static clutter components, validating the efficacy and precision of circular masking for isolating and suppressing dominant stationary reflections. Conversely, the implementation of square-region masking yielded a modest improvement, raising the SNR to −20.3 dB. Although this represented an improvement over the baseline scenario, it was less effective than the circular-region approach, suggesting that the geometric precision of masking significantly impacts clutter suppression performance.

The comparative results substantiate the proposed radar–camera fusion strategy’s capability to selectively attenuate clutter while effectively preserving signals from moving targets. By integrating spatially precise location data derived from the camera with the temporal and Doppler resolution data provided by the radar, the presented method achieves the targeted suppression of static clutter, thereby enhancing overall radar clarity and detection performance. These measurable improvements in SNR highlight the practical applicability of this integrated sensing configuration, especially in real-world scenarios characterized by low contrast or densely cluttered environments. The enhanced clarity and reduced clutter-induced false alarms greatly facilitate the reliable detection and tracking of moving targets, a critical requirement for safety-sensitive tasks such as indoor surveillance, human activity monitoring, and assistive robotics. Ultimately, the demonstrated SNR improvements directly contribute to improved accuracy, reliability, and robustness in target recognition, minimizing risks associated with misclassification or false detections.

To further substantiate the effectiveness of the proposed clutter mitigation method, an in-range detection rate analysis was performed, measuring the proportion of frames in which the detected target fell within the critical detection range from 4 to 6 m. The detection rate shows how reliably the radar system detects the target within an expected distance range [29]. The raw RD map exhibited a detection rate of 14.84%, indicative of the adverse impact of static clutter on target detectability. The application of circular-region masking significantly increased the detection rate to 23.44%, corresponding to a 57.9% relative improvement in detection performance, underscoring the method’s capability to enhance target visibility through the suppression of identified clutter regions. Square-region masking improved the detection rate to 18.75%, though its efficacy was comparatively limited. These outcomes affirm the practical advantage of clutter mitigation techniques in improving the reliability of radar-based target detection within defined spatial boundaries. Compared to traditional radar-only clutter mitigation techniques, such as MTI filtering or Doppler-based thresholding, the proposed fusion strategy demonstrates improved clutter suppression by leveraging persistent spatial cues from the camera. These cues allow for the precise masking of static clutter regions that may not be effectively filtered by Doppler-based methods alone. While direct numerical comparisons with other algorithms are beyond the scope of this study, the results indicate that integrating low-resolution visual data contributes to measurable performance improvements in both SNR and in-range detection rate. Table 1 summarizes the SNR and detection rate results of the processed RD maps.

## 4. Conclusions

In this paper, we presented a radar–camera sensor fusion methodology specifically designed for effective clutter mitigation in FMCW radar systems. By synergistically combining a 77 GHz FMCW radar and a monocular RGB camera, the proposed technique successfully suppressed static clutter in radar RD maps through visually guided adaptive region masking. Unlike traditional radar-only clutter suppression approaches, our method capitalizes on the camera sensor’s inherent capability to precisely localize persistent clutter sources, enabling targeted clutter removal while preserving critical moving target information. Experimental evaluations demonstrated that the implemented clutter removal strategies, particularly circular-region masking guided by a camera-derived spatial context, consistently improved radar signal clarity, as evidenced by measurable enhancements in the SNR. Crucially, these performance improvements were achieved without imposing significant computational overhead, highlighting the proposed approach’s suitability for real-time processing and deployment in computationally constrained environments.

The proposed framework was intentionally designed with scalability, practicality, and cost-sensitivity in mind, making it particularly suitable for deployment in safety-critical and indoor monitoring scenarios, where traditional radar systems often encounter limitations due to dense static clutter or confined spatial conditions. The strategic use of camera-driven spatial insights provides a lightweight yet powerful enhancement to existing radar signal processing workflows, significantly advancing the potential for reliable target detection and tracking in complex, clutter-intensive environments. Future research directions stemming from this work include integrating advanced learning-based clutter recognition techniques, enabling more sophisticated and context-aware masking strategies that dynamically adapt to changing environmental conditions. Furthermore, research into improved sensor alignment methodologies and calibration techniques is necessary to maximize the fusion efficiency and robustness of radar–camera systems. Finally, the implementation of the proposed framework in real-time embedded systems and its optimization for low-power operation remain essential milestones for successful deployment in practical applications such as indoor surveillance, assistive robotics, and smart-environment monitoring systems.

## Figures and Tables

**Table 1:** Performance comparison of the processed range–Doppler maps.

	Raw RD Map	With Circular-Region Masking	With Square-Region Masking
Signal-to-noise ratio	−20.7 dB	−18.7 dB	−20.3 dB
Detection rate	14.84%	23.44%	18.75%

## Data Availability

Dataset available on request from the authors.

## Abbreviations

The following abbreviations are used in this manuscript:

DNN-LSTM	Deep Neural Network—Long Short-Term Memory
FMCW	Frequency-Modulated Continuous Wave
FFT	Fast Fourier Transform
HOG	Histogram of Oriented Gradients
LiDAR	Light Detection and Ranging
MTI	Moving Target Indicator
RADAR	RAdio Detection and Ranging
ROI	Region of Interest
RGB	Red, Green, and Blue
SNR	Signal-to-Noise Ratio
